# Egg Yolk Antioxidants Profiles: Effect of Diet Supplementation with Linseeds and Tomato-Red Pepper Mixture before and after Storage

**DOI:** 10.3390/foods8080320

**Published:** 2019-08-07

**Authors:** Besma Omri, Nadir Alloui, Alessandra Durazzo, Massimo Lucarini, Alessandra Aiello, Raffaele Romano, Antonello Santini, Hedi Abdouli

**Affiliations:** 1 Laboratory of Improvement & Integrated Development of Animal Productivity & Food Resources, Higher School of Agriculture of Mateur, University of Carthage, Avenue de la République P.O. Box 77, Amilcar 1054, Tunisia; 2 National Agronomy Institute, Tunis, University of Carthage, Avenue de la République P.O. Box 77, Amilcar 1054, Tunisia; 3 Department of Veterinary Sciences, University of Batna, Batna 05000, Algeria; 4 CREA—Research Centre for Food and Nutrition, Via Ardeatina 546, 00178 Roma, Italy; 5 Department of Agriculture, University of Napoli Federico II, 80138 Napoli, Italy; 6 Department of Pharmacy, University of Napoli Federico II, Via D. Montesano 49, 80131 Napoli, Italy

**Keywords:** carotenoid, flavonoids, oxidative status, polyphenols, yolk color

## Abstract

This study evaluated the effect of dietary incorporation of linseed alone or along with dried tomato paste-pepper powder mix on egg physical characteristics, antioxidant profiles, lipid oxidative status, and yolk coloration before and after storage at 4 °C for one month. Sixty Novogen White laying hens, 27 weeks-old, were divided into three groups and given 100 g/hen/day of a standard diet (C), standard diet containing 4.5% of ground linseed (L), linseed diet containing 1% of dried tomato paste and 1% of sweet red pepper (LTP). Linseeds increased (*p* < 0.05) egg yolk antioxidant capacity but not lipid oxidative stability (*p* > 0.05). However, dietary inclusion of LTP did not improve fresh egg yolk antioxidant activity and lipid oxidation stability (*p* > 0.05). With reference to the stored eggs, only antioxidant activity measured by phosphomolybdenum reduction and lipid oxidative stability were influenced (*p* < 0.05) by the dietary treatment. Fresh egg yolk of hens fed on linseeds tended to have a slightly more yellow, redder, and less light color than the eggs of hens fed with the control diet. Dietary supplementation of LTP increased (*p* < 0.05) the Roche yolk color fan (RYCF) score and redness (a*) and decreased (*p* < 0.05) lightness (L*) without affecting (*p* > 0.05) saturation (C*). Storage of hens’ eggs fed on the control diet did not influence (*p* > 0.05) yolk color.

## 1. Introduction

The hen’s egg is considered as a functional food, since it represents a valuable source of high quality proteins, minerals, vitamins, and lipids, i.e., polyunsaturated fatty acids (PUFA) and phospholipids [1,2,3,4,5,6]. Beside their nutritional value, eggs represent a good source of antioxidants [6]: egg proteins (i.e., ovalbumin, ovotransferrin, phosvitin), phospholipids, and vitamin A, vitamin E, selenium, carotenoid, show antioxidant [7,8,9,10,11,12,13,14,15,16] and nutraceuticals properties [13,14,15,16,17,18,19,20,21,22,23,24,25,26,27].

Moreover, eggs can be also enriched with antioxidants by manipulation of poultry feed [28,29,30,31,32]. Omega-3 fatty acids and carotenoids are biologically active compounds utilized for egg biofortification [33,34] also using by-products of agro system chain in an environmentally friendly way [34,35,36,37,38,39,40].

Carotenoid [41] can be supplemented as dried synthetic pigments or as natural pigments from red pepper [42], tomato powder [36,37,43,44,45] and colored carrot [46]. They cannot be produced by hens de novo. Carotenoid must be provided in their diet’s ingredients [47]. These compounds were used as pigments for many years so as to obtain a desired color of egg yolk [48]. Egg choice by consumers is no longer only based on yolk cholesterol content or fatty acids profile, but also on its color [49] due to the health benefits associated with the pigment source [50,51,52]. As antioxidants [53], carotenoids, were used to neutralize the excess of free radicals, to protect the cell against their toxic effects and to contribute to disease prevention such as arthrosclerosis, cardiac hypertrophy, congestive heart failure and Alzheimer [54,55,56,57]. For example, according to Willcox et al. [54] beta-carotene is a strong antioxidant and is the best quencher of singlet oxygen. Lycopene, a carotenoid, has been found to be very protective, particularly for prostate cancer [55]. The current review of Bohn [56] well summarizes human observational studies/intervention trials targeting carotenoids in relation to chronic diseases characterized by oxidative stress and markers thereof: the author underlines, that, even if different markers of oxidative stress were studied, no single one gives a complete shot of oxidative stress homeostasis, related to the involvement of many body compartments, mechanisms and the type/number of bioactive compounds.

It is worth mentioning the review of Pham-Huy et al. [57] on the role of antioxidants in the prevention of chronic diseases such as cancer, autoimmune disorders, aging, cataract, rheumatoid arthritis, cardiovascular and neurodegenerative disease; antioxidants present different physiological properties, i.e., anti-allergic, antimicrobial, anti-inflammatory, anti-atherogenic, vasodilatory effects [58,59,60,61,62,63,64].

Also, the current work of Yeung et al. [65] reported, by means of a scientific literature analysis of works since 1991, that there has been a transition of scientific interest from vitamins and minerals to antioxidant phytochemicals.

Red pepper, the source of red and yellow carotenoid [66,67], used at 0.8%, increased egg yolk color scores [42]. However, dried tomato contained lycopene, the main red carotenoid responsible for egg yolk red color. The combination of red and yellow carotenoid has been found to be effective for yolk pigmentation [68]. Yellow carotenoid deposition creates a yellow base, which is necessary for the saturation of the final color. When the saturated phase was established, red carotenoid addition increased the orange-red yolk color. Furthermore, feeding hens with linseed, sunflower and palm oil accelerated egg yolk lipid oxidation, related to long chain polyunsaturated fatty acids [69].

Lipid oxidation is a process that affects egg yolk lipid stability during storage. It can alter egg nutritional quality and may lead to taste, flavor, odor and color depreciation and to toxic substances production [70,71]. Prevention of cholesterol oxidation and PUFA auto-oxidation could be reached by carotenoid supplementation, tomatoes or sweet red pepper, as sources of antioxidants into hens’ feed.

The use of linseeds, tomato by-products or red pepper as natural or biological feed additives has been done to supplement laying hens’ feeds, and consequently, the egg industry, with essential micro-ingredients (polyunsaturated fatty acids (ω-3 and ω-6) and antioxidant (α-carotene, β-carotene, β-cryptoxanthine, lycopene, zeaxanthin, total phenols and flavonoids), as well as for animal wellbeing purposes and improved efficiency. As far as red pepper is concerned, Li et al. [41] reported that the development of an environmental friendly technique which would allow the egg yolk coloring ability of red peppers to be obtained at a lower cost with enhanced efficiency could be extremely beneficial to the poultry industry; moreover, this result has led to an increase of interest of consumers.

Dietary supplementation of tomato by-products or red pepper was also a new solution to optimize livestock economic (reducing the prices of feed ingredients) and environmental performance at the industry level. This supplementation may reduce the need for synthetic antioxidants at industry level through advanced knowledge of the impact of these ingredients and their composition on animal and human health and the use of natural antioxidant to optimize the safety, sustainability and nutritional value of feed ingredients; moreover this result has led to an increase of interest from consumer.

In view of the above, the present study aims at evaluating the effect of dietary incorporation of linseed alone or along with a dried tomato paste-pepper powder mix on egg physical characteristics, egg yolk antioxidant profile, lipid oxidative status and coloration before and after storage at 4 °C for one month.

## 2. Materials and Methods

### 2.1. Ethical Considerations

All procedures concerning animals’ care, handling, and sampling were conducted under the approval of the Official Animal Care and Use Committee of the Higher School of Agriculture of Mateur (protocol N°05/15) before the initiation of the study and followed the Tunisian guidelines.

### 2.2. Experimental Design

A total of sixty, *Novogen* White laying hens (initial live weight = 1449.95 g ± 71.99 g) of 27 weeks-old were randomly regrouped into 3 homogeneous groups of 20 hens each. Corn and soybean-meal were used as a standard mash diet (C) for laying hens. Two supplemented diets were designated as follows: (1) linseed (L) and (2) linseed–tomato–pepper (LTP), which were individually prepared by mixing the control diet thoroughly with the designated supplements at the required incorporation levels, as described by Omri et al. [72] (Table 1).

To reduce the feed-selection behavior typically observed in laying hens diets were restricted to 100 g/hen/d. Feed was offered once daily at 7:30 AM. Hens were allocated individually in standard pens with individual feed-trough and common water-trough in an ambient temperature of about 20 ± 4 °C. A lighting schedule of 16 h light and 8 h dark was followed. Water was offered *ad libitum*. during the experimental period, which lasted 47 days.

### 2.3. Data Collection and Chemical Analyses

Eggs laid from the 26th to the 30th days of the experimental period were weighed and used for egg physical characteristics measurements (egg albumen, yolk and shell weights). Egg yolks were pooled per two hens belonging to the same dietary treatment group so that 10 yolk samples per group were obtained instead of 20. Yolk samples were then used for analyses: antioxidant profile, oxidative status and egg yolk color. Eggs laid during the 31st day of the experimental trial were weighted and stored at 4 °C for one month. After storage, the eggs were used for the same analyses as those conducted prior to storage.

### 2.4. Egg Yolk Color

Egg yolk color was determined using the Yolk Color Fan^®^ Scale DSM Yolk Color Fan (DSM Nutritional Products Europe, Wurmisweg 576, CH-4303 Kaiseraugst, Switzerland) (1 for light yellow and 15 for orange) and the color measuring device Konica Minolta Chroma Meter CR- 400/410 (Minolta, Tokyo, Japan) following the CIE (Commission Internationale de L’Eclairage) color system, L* (lightness: negative towards black, positive towards white) a* (redness: negative towards green, positive towards red) and b* (yellowness: negative towards blue, positive towards yellow). Chroma Meter was set up perpendicularly to the egg yolk surface in a Petri dish. L*, a* and b* parameters were measured three times. Final values were calculated as the averages of the three corresponding values measured.

Egg chroma was calculated according to the formula:

C* = (a*2 + b*2)^1/2^

### 2.5. Antioxidant Profile Determination

#### 2.5.1. Total Carotenoid Determination

Total carotenoid of fresh, stored egg yolk and diets were determined in accordance with Amaya, [73]. Samples of 0.5 g were extracted in 5 mL of Butylated Hydroxy Toluene (BHT) (0.05%) in cold acetone (4 °C) and stirred for 1 h 30 min. After 15 min of centrifugation at 3000 rpm, the supernatant was transferred to another tube containing 7 mL of petroleum ether.

Then 20 mL of distilled water was slowly added along the tube. After separation of two phases for 1 h, 10 mL of distilled water was added. The aqueous phase was discarded. The ether phase was transferred to another tube and absorbance was measured.

#### 2.5.2. Total Phenol Determination

The Folin–Ciocalteu method following the procedure of Makkar et al. [74] was used for total phenol content of acetone extracts evaluation. A total of 1 mL of acetone extract was mixed with 0.5 mL of Folin–Ciocalteu reagent, then 2.5 mL of Na_2_CO_3_ solution (20% *w*/*v*) were added. The solution was incubated for 40 min at 60 °C after vortexing and absorbance was measured at 750 nm against a blank (distilled water). Total phenolics content was expressed as mg equivalents gallic acid (EGA) (standard) per g of sample.

#### 2.5.3. Flavonoid Determination

Flavonoid content was determined by the aluminum chloride method as reported by Patel et al. [75]. Fresh, stored egg yolk or diet samples (100 mg) were extracted in 5 mL of diethyl. After centrifugation for 15 min at 2000 rpm, the precipitate was extracted in 5 mL of 80% methanol for 5 h and filtered using Wathman filter paper. The filtrate was adjusted to 50 mL with distilled water. An Aliquot of 2.5 mL was mixed with 0.15 mL NaNO_2_ (5%). After 5 min, 0.15 mL of aluminum chloride (10%) was added. Six minutes later, 1 mL of NaOH (1N) and 1.2 mL of distilled water were added. The solution was vortexed and absorbance was measured at 510 nm against distilled water (blank). The flavonoid content was expressed as mg equivalents catechin (standard) per g of sample.

#### 2.5.4. Oxidative Status Determination

Antioxidant activity [76,77] of fresh and stored egg yolk was measured by the phosphomolybdenum method according to the procedure described by Prieto et al. [78]. A total of 0.5 g of fresh egg yolk was diluted in 10 mL of NaCl (2%). A total of 100 µL of the suspension was adjusted to 2 mL with distilled water. Then, 2 mL of phosphomolybdenum reagent (2.8 mM of sodium phosphate and 4 mM ammonium molybdate in 0.6 M sulphuric acid) was added. After incubation at 95 °C for 90 min., the mixture was cooled at room temperature and the absorbance was measured at 695 nm (A_695 nm_) using a UV-visible spectrophotometer (CECIL Auruis series CE 2041 UV/Vis) against 2% NaCl (blank). Antioxidant capacity is expressed as mg equivalents of ascorbic acid (EAA) (standard) per g of sample.

Total antioxidant activity was also evaluated by ferric reducing antioxidant power assay, according to Benzie and Szeto [79], with slight modifications. Aqueous solutions of egg yolk prepared as reported above were used for this assay. A total of 150 µL of solution was mixed with 2.4 mL of distilled water, 0.45 mL of ethanol, 0.75 mL of HCl, 0.75 mL of 1% potassium ferricyanide, 0.25 mL of 1% SDS and 0.25 mL of 0.2% ferric chloride. The solution was left in a water bath at 50 °C for 20 min then cooled at room temperature and absorbance was measured at 750 nm. Antioxidant capacity is expressed as mg Equivalents Gallic Acid (EGA) (standard) per g of sample.

The malondialdehyde (MDA) measurement method described by Draper and Hadeley [80] was used for lipid oxidation of fresh and stored egg yolk estimation. A total of 0.5 g of egg yolk was diluted in 5 mL of TCA (20%) with 0.5 mL of BHT (1%) in absolute ethanol. After stirring for 1 h at room temperature, samples were centrifuged for 15 min at 3000 rpm. A total of 2.95 mL of thiobarbituric acid (TBA) (50 mM) were added to 1.5 mL of solution. After incubation at 100 °C for 10 min, the samples were cooled at room temperature and centrifuged at 3500 rpm for 10 min. Absorbance was measured at 532 nm using an UV-visible spectrophotometer against 2% NaCl (blank). Thiobarbituric acid was used as a standard. The thiobarbituric acid reactive substances (TBARS) are expressed as µg of MDA per g of sample.

### 2.6. Statistical Analysis

Data were tested for ‘diet’ (C, L and LTP) and ‘type of eggs’ (fresh or stored) effects and their interaction using mixed models with compound symmetry covariance structures of SAS [81]. Interaction ‘diet x type of eggs’ effects were not so significant for all parameters that comparisons of intra-diet means/egg type (C versus L and L vs. LTP for fresh and stored eggs separately) and intra-type of eggs/diet (fresh versus stored eggs for each diet) were not possible. Eggs were also stored without separation between yolk, albumen and shell so that all parameters were not determined on the same eggs before and after storage, but separately, on representative samples of eggs laid/diet.

Data were tested for ‘diet’ (C, L and LTP) using the GLM procedure (General Linear Model) of SAS according to the following model:

Y_ij_ = µ + T_i_ + e_ij_

where:

Y_ij_ = represents the jth observation on the ith treatment
µ = overall mean
T_i_ = the main effect of the ith treatment
e_ij_ = random error present in the jth observation on the ith treatment

When diet and/or egg type effects were significant at α < 0.05, means comparisons were tested using the CONTRAST statement of SAS [82]. The effect of storage on eggs/diet was tested using the Student T-test. Correlations between egg yolk color traits and carotenoid concentration were tested using the procedure CORR of SAS [81]. All statistical procedures were tested using SAS [81].

## 3. Results and Discussion

### 3.1. Egg Physical Characteristics

Egg physical characteristics before and after storage at 4 °C for 30 days are reported in Table 2.

As reported in our previous research study [72], dietary supplementation of 4.5% of linseeds increased (*p* > 0.05) fresh egg weight, and compared to the linseeds (L), tomato-sweet pepper mixture was without effect (*p* > 0.05). However, stored egg weight was influenced (*p* < 0.05) with dietary treatment and, for each of the four treatments, there was a slight loss of egg weight after storage at 4 °C. Fresh and stored egg yolk weights were not influenced (*p* > 0.05) by dietary treatment. Dietary treatment did not affect (*p* > 0.05) the fresh eggs’ albumen weight. However, LTP increased (*p* < 0.05) the stored eggs’ albumen weight. Storage decreased (*p* < 0.05) albumen weight of hens fed with the control diet. The shell weight of fresh eggs was not affected (*p* > 0.05) by dietary treatment, but increased (*p* < 0.05) by L and LTP addition for stored eggs. Storage decreased (*p* < 0.05) egg shell weight of hens fed on the LTP diet. Shell thickness was affected (*p* > 0.05) neither by dietary treatment nor by storage. In this regard, Yassein et al. [83] showed that dietary addition of 5% of linseeds did not affect egg weight and albumen, yolk and shell percentages. Ahmad et al. [84] found that feeding hens with 5% of linseed did not affect egg weight (59.93 g vs. 60.47 g), yolk weight (15.15 g vs. 14.81 g), albumen weight (9.05 g vs. 10.51 g), shell weight (8.13 g vs. 8.48 g) and shell thickness (0.39 g vs. 0.38 mm).

Concerning tomato, Akdemir et al. [43] reported that the dietary supplementation of 0.5% and 1% of tomato powder did not influence yolk weight (16.59 g vs. 17.11 g), shell weight (6.79 g vs. 6.97 g) and shell thickness (0.399 mm vs. 0.393 mm).

Studies on the effect of egg storage on its physical characteristics are lacking. However, Niemiec et al. [85] reported that the dietary addition of primrose, linseeds and rapeseeds at, respectively, 2.88%, 3.66% and 5%, with or without supplementation of 200 mg vitamin E/kg, did not affect egg weight after 20 days of storage at 12 °C. A significant reduction in the egg yolk weight after storage was found, from 24.23% (control group) to 23.35% (primrose, linseeds and rapeseeds) and 23.18% (primrose, linseeds, rapeseeds and vitamin E).

### 3.2. Egg Yolk Antioxidant Profile

Egg yolk antioxidants [86] (α-carotene, β-carotene, β-cryptoxanthine, lycopene, zeaxanthine, total phenols and flavonoids) concentrations before and after storage at 4 °C for 30 days are reported in Table 3.

Fresh eggs concentrations of all antioxidants were affected (*p* < 0.05) by dietary treatment. Carotenoid concentrations of fresh eggs of hens fed with control diet (C) varied from 7.7 to 12.4 μg/g of yolk, respectively, for lycopene and zeaxanthine [72]. These levels were in agreement with those (12.8 and 9.2 μg/g DM, respectively, for luteine and zeaxanthine) reported by Englmaierovà et al. [49] of eggs from hens fed with corn, wheat, soybean meal and alfalfa meal. Our results, were higher than those reported by Hammershøj et al. [46] who found that eggs of hens fed on a standard organic food (wheat, oats, peas, sunflower meal, fish meal) contained: 7.46 μg/g of lutein, 2.6 μg/g of zeaxanthine, 0.01 μg/g of α-carotene/g and 0.03 μg/g of β-carotene. Our results were also higher than those (7.09 μg/g of luteine, 0.85 μg/g of cis-luteine, 7.09 μg/g of zeaxanthine, 0.69 μg/g of cis-zeaxanthine and 1.07 μg/g of β-carotene) reported by Kotrbáček et al. [87] for a diet containing 33% of wheat, 30% of corn and 24% of soybean meal. Studies in the literature with regard to this aspect are lacking. However, a total phenol content equal to 0.54 mg GAE/g DM was reported by Amar et al. [88] and equal to 0.72 mg GAE/g (diet based on yellow corn) and 0.66 mg GAE/g DM (diet based on wheat) were found by Nimalaratne et al. [14]. Dietary incorporation of linseeds increased (*p* < 0.05) fresh egg yolk concentrations of carotenoid and total phenols and did not affect (*p* > 0.05) egg yolk concentration of flavonoids.

Diet with dried tomato (1%) and sweet red pepper (1%) mix contained four times more carotenoid than diet with linseeds (L). However, fresh egg yolk carotenoid concentrations of hens fed with L and LTP were not different (*p* > 0.05). Studies on the effects of linseeds on the egg yolk antioxidants profile are lacking. Concerning tomato, Habanabashaka et al. [89] reported that dietary incorporation of 0, 3, 6 or 9% of tomato by-products increased egg yolk concentration of lycopene from 0.01 to 0.95 µg/g, lutein from 10.1 to 13.9 µg/g and of zeaxanthine from 9.4 to 12.9µg/g. However, Amar et al. [88] reported that dietary incorporation of 0, 4, 7, 10 and 13% of tomato peel increased egg yolk concentration of lycopene from 26.5 µg/g DM (4%) to 42.8 µg/g DM (7%); 37.6 µg/g DM (10%) and 41.8 µg/g DM (13%) and of β-carotene from 6.5 µg/g DM (0%); 11.3 (4%) µg/g DM; 17.6 (7%) µg/g DM; 12.3 (10%) µg/g DM to 16.7 µg/g DM (13%). Akdemir et al. [43] evaluated the effect of the dietary addition of 0.5%, 1% of tomato powder reported that an increase in egg yolk concentration of lycopene from 6.53 (0.5%) to 8.05 µg/g (1%), of β- carotene form 172 µg/g (0%) to 331 µg/g (0.5%) and 551 µg/g (1%) and of lutein from 6.85 µg/g (0%) to 7.23 µg/g (0.5%) and 9.03 µg/g (1%). Dietary incorporation of 0.1%, 0.2%, 0.4%, 0.8% of paprika extract increased egg yolk concentration of total carotenoid from 3.43 μg/g (0%) to 7.7 μg/g (0.1%), 10.86 μg/g (0.2%), 14.60 μg/g (0.4%) and 16.83 μg/g (0.8%) [90]. Egg storage for one month did not affect (*p* > 0.05) the egg yolk concentrations of all carotenoids (α-carotene, β-carotene, cryptoxanthin, lycopene and zeaxanthin) and decreased (*p* < 0.05) the total phenol concentrations of each treatment.

Reports on the effect of storage of eggs of hens fed with similar treatments to ours diets are lacking. However, Barbarosa et al. [91] reported that egg storage for 35 days at room temperature (26.5 °C) reduced egg yolk total carotenoid concentration from 28.55 to 22.09 μg/g. By contrast, egg storage for 35 days at 7.9 °C reduced total carotenoid egg yolk concentration from 28.55 to 23.57 μg/g. Gawecki et al. [92] also reported that egg storage for 8 weeks at 2 °C did not reduce egg yolk total carotenoid concentration. However, after 15 weeks of storage, the total carotenoid concentration in the egg yolk decreased from 28.55 µg/g to 27.03 μg/g.

In the present study, egg yolk antioxidant capacity was evaluated by determining egg yolk capacity to reduce MO^6+^ to MO^5+^ and Fe^3+^ to Fe^2+^. Lipid oxidative stability determined by thiobarbituric acid reactive substances (TBARS) was even higher than the concentration of MDA, which was low.

Only fresh egg antioxidant activity determined by the reduction of MO^6+^ to MO^5+^ was influenced (*p* < 0.05) by dietary treatment. Fresh egg antioxidant activity of the control group was equal to 4.48 mg AAE/g. Reports in the literature concerning egg yolk antioxidant activity expressed as AAE and as GAE are lacking. Fresh egg yolk concentration of MDA was not affected (*p* > 0.05) by dietary treatment. Fresh egg yolk concentration of MDA of the control treatment was equal to 0.11 μg/g [72]. This value was lower than the 1.17 μg/g reported by Englmaierovà et al. [49] and the 0.7 μg/g reported by Venglovská et al. [93] for fresh eggs of hens fed on corn, wheat and soybean meal. Our results were similar to those reported by Hayat et al. [94] with a mean value of 0.1 μg/g for fresh eggs of hens fed on corn meal and soybean meal.

The dietary addition of 4.5% of linseeds increased (*p* < 0.05) the egg yolk antioxidant capacity, but not its lipids oxidative stability (*p* > 0.05). Thus, egg enrichment with fatty acids (polyunsaturated and polyunsaturated ω-3) through the dietary supplementation of linseeds did not reduce egg yolk lipid oxidative stability. This lipid protection against oxidation may be attributed to the increase of fresh egg yolk pigments and total phenol concentrations (Table 3). In this regard, Hayat et al. [94] reported that egg yolk MDA concentration increased from 0.1 to 0.23 μg/g (10% of linseeds flaxseed), 0.32 μg/g (10% of linseeds plus 50 IU of α-tocopherol) and 0.28 μg/g (10% of linseeds plus 150 mg of BHT). However, Boruta and Niemiec [95] reported that the dietary addition of 3% of linseeds with or without supplementation of 200 mg/kg of vitamin E did not affect fresh egg yolk concentration of thiobarbituric acid-reactive substances (TBARS). The dietary inclusion of 1% of dried tomato and 1% of sweet red pepper in addition to the linseeds did not improve fresh egg yolk antioxidant activity and its lipid oxidative stability (*p* > 0.05). This inefficiency of dried tomato and sweet red pepper mixture could be due to its low level of incorporation (2%) that did not affect egg yolk concentrations of pigments, total phenols and flavonoids when compared to diet with linseeds (Table 4).

Akdemir et al. [43] showed that the dietary addition of 0.5% and 1% tomato powder reduced egg yolk concentration of MDA from 0.33 µg/g (control) to 0.25 µg/g (0.5%) and 0.21 µg/g (1%). Sahin et al. [96] also reported the dietary addition of 100 and 200 mg of lycopene/kg in Japanese quail diet reduced the egg yolk concentration of MDA from 0.86 (control) to 0.79 µg/g (100 mg of lycopene/kg) and 0.74 μg/g (200 mg of lycopene/kg). Dietary addition of carophyll, lutein or algae (chlorella) improved egg yolk the oxidative stability from 1.17 μg/g (control) to 1 μg/g (carophyll), 0.87 μg/g (lutein) and 0.90 mg/kg (algae) [49].

Concerning stored eggs, only antioxidant activity measured by phosphomolybdenum reduction and lipid oxidative stability were influenced (*p* < 0.05) by dietary treatment. Diet with linseeds (4.5%) plus sweet red pepper and tomato mix (2%) was associated with higher (*p* < 0.05) lipid oxidative stability than diet with linseeds. However, egg storage decreased (*p* < 0.05) yolk antioxidant activity but not lipid stability to the oxidation of the four treatments. In this regard, Pereira [97] reported that MDA egg yolk concentration increased from 0.52 to 0.71 and 0.90 μg/g after storage at 4 °C for, respectively, 60 and 90 j. By contrast, Hayat et al. [94] reported that egg storage at 4 °C for 20, 40 and 60 days did not affect MDA concentration. Shahryar et al. [70] reported that ω-3 and ω-6 enriched eggs stored at 4 °C for 30 and 60 days increased MDA concentrations. Boruta and Niemiec [95] evaluated the effect of dietary addition of 4% rapeseed, 3% linseeds and 2% primrose, with or without supplementation of 200 mg/kg of vitamin E, on egg antioxidant status and reported that egg storage for 3 and 6 months increased yolk MDA concentration. However, vitamin E supplementation decreased egg yolk MDA concentration after 6 months of storage.

### 3.3. Egg Yolk Coloration

Egg yolk coloration before and after storage at 4 °C for one month are represented in Table 5.

Color scores determined by the Yolk Color Fan^®^ scale (RYCF) were affected by dietary treatment (*p* < 0.0001). Fresh egg yolk score of the control group was the lowest with a mean value of 4.67 [72]. Our values were not in agreement with 6.65 reported by Abdouli et al. [98] and 8.64 found by Lokaewmanee et al. [99] for eggs of hens fed with a similar diet to our control treatment. b* mean value showed that egg yolk yellow color was sufficiently intense. Our results were lower than 32.5 and 48.2 reported, respectively by, Abdouli et al. [98] and Dvorak et al. [100]. Negative mean value of a* indicated an absence of the red hue. Higher mean values ranging from 0.05 to 13.5 were reported by Dvorak et al. [100]. L* and C* mean values showed that the egg yolks of hens fed with control treatment were characterized by an intense, light yellow color. Dietary incorporation of 4.5% of linseeds increased (*p* < 0.05) the egg yolk color score (RYCF). The fresh egg yolk of hens fed with linseeds tended to have a slightly more yellow, redder and less light color than the eggs of the control group. Correlations between the yolk color scores (RYCF), parameters determined by Chroma Meter and pigments concentrations (Table 6), showed that the yolk color scores (RYCF) and redness (a *) were positively correlated with all the determined pigments.

However, lightness (L*) and yellowness (b*) were negatively correlated with all the determined pigments. Dietary supplementation of the tomato and the red pepper mixture (2%) increased (*p* < 0.05) the RYCF score and the red hue (a*), and decreased (*p* < 0.05) the lightness (L*) without affecting (*p* > 0.05) the saturation (C*). These results are in agreement with those reported by Akdemir et al. [43] who found that dietary supplementation of 0.5% or 1% of dried tomato increased egg yolk color from 11.25 (control) to 13.08 (0.5%) and 13.58 (1%). The dietary addition of 130 g of dried tomato peel/kg DM increased the egg yolk color index from 8.5 to 14.6 [88]. Habanabashaka et al. [89] reported that the supplementation of 6% of tomato waste meal increased egg yolk scores from 4.66 (control) to 9.15 (6%). Salajedheh et al. [101] found that dietary incorporation of 19% of dried tomato pomace had a significant effect on egg yolk color scores which increased from 7.25 to 9.38. Dietary addition of 50 and 100 g/kg of dried tomato powder increased egg yolk color from 6.7 (control) to 9.7 and 10.3 for respectively, 50 and 100 g/kg of dried tomato powder [102]. Salajedheh et al. [101], also reported that the dietary supplementation of 150 and 190 g/kg of dried tomato powder increased egg yolk color from 7.25 (control) to, respectively, 8.50 (150 g/kg) and 9.83 (190 g/kg). Studies on the effect of red pepper powder on the egg yolk color are lacking. However, Niu et al. [90] showed that the dietary addition of 0.8% of paprika increased egg yolk color from 1.7 to 9.9. Li et al. [42] also reported that dietary supplementation of 0.3 to 4.8 or 9.6 ppm and 0.8% of red pepper powder increased egg yolk color from 7.7 to 12.7.

Studies on the effect of linseeds on egg yolk color are lacking. However, Yassein et al. [83] showed a decrease in yolk color related to linseed supplementation at a level of 5%. Reports on L*a*b* color space in response to dietary supplementation of carotenoid are also lacking.

Concerning stored eggs, with the exception of saturation (C*), all other parameters were influenced (*p* < 0.05) by dietary treatment. Storage of eggs of hens fed with the control diet (C) did not affect (*p* > 0.05) the yolk score and color parameters determined by colorimetry. By contrast, the storage increased (*p* < 0.05) yolk lightness (L*) and decreased redness (a*) of eggs corresponding to linseeds treatment (L). However, stored eggs corresponding to the LTP- treatment were found to be less colorful (lower RYCF score) and lighter (higher L*) than fresh eggs. Studies on the effect of the storage on egg yolk color are lacking. However, Barbarosa et al. [91] reported that storage of ω-3 enriched eggs for 35 days with or without refrigeration decreased the yolk color. This decrease became significant from the 28th day of storage for eggs stored at room temperature (26.5°). For eggs stored at 7.9 °C, egg yolk color reduction was not significant during the 35 days.

The use of dried tomato and red pepper as natural or biological antioxidants has been a new solution to reduce the supplementation of synthetic pigments as feed additives in laying hens’ diets. However, the stability of these antioxidants may be affected over a long period of storage. In fact, degradation reactions (chemical, enzymatic and physical) can cause undesirable changes in the appearance, color and texture as well as in the nutrient content of the laying hens’ diets. These reactions can be responsible for pigment losses during storage. These losses must be considered when formulating feed in order to avoid complaints. Also, the quality of these supplements may be affected by several factors such as tomato and pepper variety, soil, cultural practices and climate.

## 4. Conclusions

The results of this study clearly show how dietary inclusion of 4.5% of linseed significantly increased egg yolk concentrations of antioxidants profile. This dietary supplementation did not increase yolk concentration of MDA before and after storage at 4 °C for one month. Enriching linseed-supplemented feed with 1% tomato and 1% sweet red pepper did not enhance egg physical characteristics, yolk color, antioxidants profile and its lipid oxidative stability.

## Figures and Tables

**Table 1:** Ingredients, chemical and antioxidants composition of the experimental diet (g/Kg) *.

			Diets
	Control (C)	Linseeds (L)	Linseeds–Tomato–Pepper (LTP)
**Ingredients (%)**			
Linseed	0	4.5	4.5
Dried Tomato	0	0	1
Sweet red pepper	0	0	1
Yellow corn	66.5	63.5	61.5
Soybean meal	25.5	24.0	24.0
Calcium carbonate, Mineral and Vitamin mixture	8.0	8.0	8.0
**Chemical Composition**			
Crude protein, (%, dry matter (DM))	18.1	18	18
Ether extract (%, DM)	3.56	5.6	5.27
Metabolizable energy (Kcal/Kg DM)	2750	2850	2830
**Antioxidants**			
α-carotene, * (10^−9^) g/kg DM	3.41	5.1	21.7
β-carotene, * (10^−9^) g/kg DM	3.37	5.36	23.2
β-cryptoxanthine,* (10^−9^) g/kg DM	3.84	5.50	25.3
Lycopene, * (10^−9^) g/kg DM	1.77	3.48	15.7
Zeaxanhine, * (10^−9^) g/kg DM	3.90	5.59	25.7
^£^ Flavonoids, g CE/kg DM	2.26	1.59	2.03
^¥^ Total phenols, g GAE/kg DM	3.02	3.53	2.98

* Note: C = Control diet; L = diet supplemented with ground linseed at 4.5%, LTP = diet supplemented with ground linseed (4.5%), dried tomato paste (1%) and sweet pepper powder (1%) mix; ^¥^  Total phenols expressed in g gallic acid equivalent, g GAE/kg DM; ^£^ Flavonoids expressed in g catechin equivalent, g CE/kg DM. Control (C) provided following nutrients per 100 g: Ca, 4.3 g; P, 0.6 g; Na, 0.14 g; Cl, 0.23 g; Fe, 4 mg; Zn, 40 mg; Mn, 7 mg; Cu, 0.3 mg; I, 0.08 mg; Se, 0.01 mg; Co, 0.02; methionine, 0.39 g; methionine + cysteine, 0.69 g; lysine, 0.89 g; Retinol, 800 IU; Cholecalciferol, 220 IU; α-tocopherol, 1.1 IU; Thiamin, 0.33 IU; Nicotinic acid, 909 IU.

**Table 2:** Physical characteristics of eggs before and after storage at 4 °C for one month.

Parameters	Eggs	Diets			*p*-Value
		C ^α^	L ^α^	LTP ^α^	
Egg weight, g	Fresh	55.48 ^aA^	57.67 ^aA^	57.09 ^aA^	0.07
	Stored	54.08 ^cA^	56.87 ^aA^	56.21 ^abA^	0.024
Yolk weight, g	Fresh	13.79 ^aA^	13.84 ^aA^	13.86 ^aA^	0.99
	Stored	14.54 ^aA^	14.69 ^aB^	14.48 ^aB^	0.92
Albumen weight, g	Fresh	33.95 ^aA^	35.53 ^aA^	34.73 ^aA^	0.13
	Stored	31.88 ^bB^	34.00 ^aA^	33.68 ^aA^	0.046
Shell weight, g	Fresh	5.4 ^aA^	6.15 ^aA^	5.89 ^aA^	0.16
	Stored	5.2 ^cA^	5.65 ^aA^	5.58 ^abB^	0.004
Shell thickness, mm	Fresh	0.39 ^aA^	0.4 ^aA^	0.42 ^aA^	0.15
	Stored	0.4 ^aA^	0.42 ^aA^	0.41 ^aA^	0.41

Note: ^α^ C = Control diet; L = diet supplemented with ground linseed at 4.5%, LTP = diet supplemented with ground linseed (4.5%), dried tomato paste (1%) and sweet pepper powder (1%) mix; ^a,b,c^: Means within the same row with no different superscripts letters are not significantly different (*p* > 0.05); ^A,B^: Means of the same parameters within the same column with no different superscripts letters are not significantly different (*p* > 0.05); Data for fresh eggs from Omri et al. [72].

**Table 3:** Egg yolk antioxidants profile before and after storage at 4 °C for one month.

Parameters	Eggs	Diets			*p*-Value
		C ^α^	L ^α^	LTP ^α^	
α-carotene, µg/g	Fresh	11.0 ^bA^	12.26 ^aA^	12.7 ^aA^	0.0002
	Stored	11.47 ^abA^	11.66 ^aA^	12.03 ^aA^	0.033
β-carotene, µg/g	Fresh	11.2 ^bA^	12.3 ^aA^	12.9 ^aA^	0.0001
	Stored	11.53 ^aA^	11.71 ^aA^	12.18 ^aA^	0.06
β-Cryptoxanthine, µg/g	Fresh	12.4 ^bA^	13.81 ^aA^	14.42 ^aA^	0.0001
	Stored	12.59 ^aA^	13.11 ^aA^	13.45 ^aA^	0.089
Lycopene, µg/g	Fresh	7.67 ^bA^	8.42 ^aA^	8.90 ^aA^	<0.0001
	Stored	7.98 ^bA^	8.09 ^aA^	8.37 ^aA^	0.034
Zeaxanthine, µg/g	Fresh	12.4 ^bA^	13.81 ^aA^	14.42 ^aA^	0.0001
	Stored	12.59 ^aA^	13.11 ^aA^	13.45 ^aA^	0.089
Total phenols, mg GAE/g ^¥^	Fresh	1.86 ^bA^	2.17 ^aA^	2.16 ^aA^	0.0034
	Stored	1.57 ^aB^	1.74^aB^	1.64 ^aB^	0.69
Flavonoids, mg CE/g ^£^	Fresh	1.92 ^bA^	1.53 ^bA^	2.96 ^aA^	0.0009
	Stored	1.50 ^aA^	1.39 ^aA^	2.17 ^aA^	0.38

Note: ^α^ C = Control diet; L = diet supplemented with ground linseed at 4.5%, LTP = diet supplemented with ground linseed (4.5%), dried tomato paste (1%) and sweet pepper powder (1%) mix; ^¥^: Total phenols expressed in mg gallic acid equivalent, mg GAE/g; ^£^: Flavonoids expressed in mg catechin equivalent, mg CE/g; ^a,b,c^: Means within the same row with no different superscripts letters are not significantly different (*p* > 0.05); ^A,B^: Means of the same parameters within the same column with no different superscripts letters are not significantly different (*p* > 0.05); data for fresh eggs from Omri et al. [72].

**Table 4:** Egg yolk lipid oxidative status before and after storage at 4 °C for one month.

Parameters	Eggs	Diets			*p*-Value
		C ^α^	L ^α^	LTP ^α^	
Antioxidant activity, mg AAE/g ^§^	Fresh	4.48 ^bA^	5.07 ^aA^	5.16 ^aA^	0.0009
	Stored	3.68 ^aB^	3.89 ^aB^	3.99 ^aB^	0.0004
Antioxidant activity, mg GAE/g ^¥^	Fresh	3.14 ^aA^	4.38 ^aA^	4.14 ^aA^	0.11
	Stored	1.16 ^aB^	1.40 ^aB^	1.58 ^aB^	0.16
Thiobarbituric acid reactive substances (TBARS), μg MDA/g	Fresh	0.11 ^aA^	0.14 ^aA^	0.15 ^aA^	0.28
	Stored	0.14 ^bA^	0.24 ^aA^	0.16 ^bA^	0.01

Note: ^α^ C = Control diet; L = diet supplemented with ground linseed at 4.5%, LTP = diet supplemented with ground linseed (4.5%), dried tomato paste (1%) and sweet pepper powder (1%) mix; ^§^: Antioxidant activity evaluated as phosphomolybdenum reducing power and expressed in ascorbic acid equivalent (AAE); ^¥^: Antioxidant activity evaluated as ferric reducing power and expressed in gallic acid equivalent (GAE); ^a,b,c^: Means within the same row with no different superscripts letters are not significantly different (*p* > 0.05); ^A,B^: Means of the same parameters within the same column with no different superscripts letters are not significantly different (*p* > 0.05); data for fresh eggs from Omri et al. [72].

**Table 5:** Egg yolk coloration before and after storage at 4 °C for one month.

Parameters	Eggs	Diets			*p*-Value
		C ^α^	L ^α^	LTP ^α^	
RYCF	Fresh	4.67 ^cA^	5.65 ^bA^	8.2 ^aA^	<0.0001
	Stored	4.7 ^cA^	5.4 ^bA^	7.53 ^aA^	<0.0001
L*	Fresh	72.65 ^aA^	71.63 ^aA^	69.42 ^bA^	<0.0001
	Stored	73.32 ^aA^	72.84 ^aB^	70.47 ^bB^	0.003
a*	Fresh	−0.59 ^bA^	1.19 ^bA^	6.59 ^aA^	<0.0001
	Stored	0.59 ^bA^	0.53 ^bB^	6.44 ^aA^	<0.0001
b*	Fresh	62.81 ^aA^	64.91 ^aA^	60.28 ^aA^	0.1
	Stored	64.59 ^aA^	64.06 ^aA^	61.22 ^bA^	0.003
C*	Fresh	62.85 ^aA^	64.95 ^aA^	60.70 ^aA^	0.15
	Stored	64.46 ^aA^	64.07 ^aA^	61.58 ^aA^	0.06

Note: ^α^ C= Control diet; L = diet supplemented with ground linseed at 4.5%, LTP = diet supplemented with ground linseed (4.5%), dried tomato paste (1%) and sweet pepper powder (1%) mix; RYCF: Roche Yolk Color Fan; ^a,b,c^ Means within the same row with no different superscripts letters are not significantly different (*p* > 0.05); ^A,B^ Means of the same parameters within the same column with no different superscripts letters are not significantly different (*p* > 0.05); data on fresh eggs from Omri et al. [72].

**Table 6:** Correlation between fresh and stored egg yolk coloration and carotenoid concentration.

Parameters	Eggs	α-Carotene	β-Carotene	β-Cryptoxanthine	Lycopene	Zeaxanthine
RYCF	Fresh	0.53 **	0.55 **	0.52 **	0.57 ***	0.52 **
	Stored	0.41 *	0.39 *	0.34 *	0.40 *	0.34 *
L*	Fresh	−0.56 **	−0.54 **	−0.47 *	−0.58 ***	−0.47 *
	Stored	−0.09	−0.29	−0.24	−0.09	−0.24
a*	Fresh	0.54 **	0.52 **	0.47 *	0.57 ***	0.47 *
	Stored	0.38 *	0.37 *	0.30	0.38 *	0.30
b*	Fresh	−0.12	−0.17	−0.018	−0.13	0.018
	Stored	0.13	0.07	0.02	0.12	0.02
C*	Fresh	−0.10	−0.15	0.003	−0.115	0.003
	Stored	0.15	0.09	0.04	0.14	0.04

*** = *p* < 0.0001, ** = *p* < 0.001, * = *p* < 0.05.
